# 

**Published:** 1860-12

**Authors:** 


### DENTAL REGISTER

### OF THE WEST.

EDITED BY

J. TAFT AND GEO. WATT.

DECEMBER–1860.

**MONTHLY:**

**Three Dollars per Annum, in advance.**

CINCINNATI:

J. T. TOLAND, PUBLISHER AND PROPRIETOR.

J. Taft, Dentist, No. 56 West Fourth Street.

